# Impact of Tuberculosis on Disease Severity and Viral Shedding Duration in COVID-19 Patients

**DOI:** 10.3390/v16020260

**Published:** 2024-02-06

**Authors:** Wei Huang, Ping Liu, Bo Yan, Fang Zheng, Yang Yang, Xiuhong Xi, Lu Xia, Yinzhong Shen

**Affiliations:** 1 Department of Tuberculosis, Shanghai Public Health Clinical Center, Fudan University, Shanghai 201508, China; 2 Center for Tuberculosis Research, Shanghai Public Health Clinical Center, Fudan University, Shanghai 201508, China; 3 Department of Medical Affairs, Shanghai Public Health Clinical Center, Fudan University, Shanghai 201508, China; 4 Department of Infection and Immunity, Shanghai Public Health Clinical Center, Fudan University, Shanghai 201508, China

**Keywords:** COVID-19, tuberculosis, severity, viral shedding

## Abstract

Accumulating evidence show a potential association between tuberculosis and COVID-19 disease severity. To further clarify the impact of tuberculosis on COVID-19 disease severity and viral shedding duration, a retrospective study was conducted on 223 COVID-19 patients, including 34 with tuberculosis and 189 without tuberculosis. Clinical information and viral load shedding time were collected. A higher percentage of severe/critical COVID-19 diagnosis and deaths was observed in patients with tuberculosis than in those without tuberculosis (8.8% vs. 3.2%, *p* = 0.142; 2.9% vs. 1.1%, *p* = 0.393), and COVID-19 patients with tuberculosis had longer viral shedding than those without tuberculosis (median: 15.0 days vs. 11.0 days; *p* = 0.0001). Having tuberculosis (HR = 2.21, 95% CI 1.37–3.00; *p* = 0.000), being of elderly age (HR = 1.02, 95% CI 1.01–1.03; *p* = 0.001) and being diagnosed with severe or critical COVID-19 (HR = 5.63, 95% CI 2.10–15.05; *p* = 0.001) were independent factors associated with prolonged virus time of SARS-CoV-2. COVID-19 patients with tuberculosis receiving anti-tuberculosis therapy time (ATT) for <2 months had a significantly longer virus shedding duration than those receiving ATT for ≥ 4 months (17.5 vs. 11.5 days, *p* = 0.012). Our results demonstrated that COVID-19 patients with tuberculosis tend to have more severe disease and a worse prognosis, and tuberculosis prolonged viral shedding, highlighting special attention and/or care required for COVID-19 patients with tuberculosis receiving ATT for <2 months.

## 1. Introduction

COVID-19 was declared a global pandemic and public health emergency by the WHO on 11 March 2020. By 2 August 2023, there were more than 768 million confirmed cases of COVID-19 worldwide, including approximately 6.9 million deaths [1]. Currently, a number of risk factors have been identified to have a potential impact on increasing its severity and mortality, including old age, being of the male sex, and pre-existing comorbidities [2]. In addition, coinfected (such as influenza–COVID-19 coinfection, and SARS-CoV-2 infection among people living with HIV) patients have been shown to be at an elevated risk for poor outcomes compared to mono-infected COVID-19 patients [3,4]. In addition to COVID-19, tuberculosis is still considered to be a major infectious disease, with 10.6 million newly diagnosed cases and 1.6 million deaths in 2021 [5]. Amid the COVID-19 epidemic, several countries have reported tuberculosis–COVID-19 coinfection through some studies [6,7,8], and increasing numbers of tuberculosis–COVID-19 coinfection cases have been reported. Several reviews and meta-analyses have shown that COVID-19 patients with tuberculosis have a higher risk of death or poorer therapeutic outcomes compared to counterparts without tuberculosis [9,10,11,12].

The SARS-CoV-2 viral load and shedding kinetics are crucial determinants of infectivity and transmission, and affect its treatment and prognosis. Risk factors for viral shedding affect the development of effective and stepped therapeutic strategies and successful public prevention policies, especially for countries with limited medical resources. A previous study reported that prolonged SARS-CoV-2 RNA detection was associated with demographic factors such as older age, being of the male sex, symptomatic status, and having received fewer doses of vaccination [13]. In addition, a recent study demonstrated that about half of immunocompromised patients with transplant recipients or hematologic malignancies shed viable virus for >4 weeks, and B cell depletion was consistently associated with prolonged viral shedding, but the use of COVID-19-specific therapies (e.g., Remdesivir, Nirmatrelvir/ritonavir) was not associated with the duration of viral shedding [14]. However, the impact of tuberculosis on the viral shedding of SARS-CoV-2 remains less known; particularly, the effects of the duration of anti-TB treatment on the outcomes and viral shedding in COVID-19 patients with tuberculosis have hardly been reported in previous publications.

Therefore, this study aimed to reveal the impact of active tuberculosis on COVID-19 disease severity and viral shedding in COVID-19 patients with tuberculosis. 

## 2. Methods

### 2.1. Study Patients

A retrospective, single-center cohort study was conducted on patients admitted to Shanghai Public Health Clinical Center, Fudan University. The patients evaluated include patients infected with COVID-19 and with tuberculosis (from February 2020 to June 2021), and patients infected with COVID-19 without tuberculosis (from April 2020 to June 2020). The patients were diagnosed with COVID-19 according to the China national guideline for COVID-19 diagnosis and treatment [15]. The tuberculosis patients were defined as bacteriologically confirmed or clinically diagnosed cases of TB.

### 2.2. Data Collection

Demographic data, comorbidities, vaccination status, location of tuberculosis lesions, clinical presentation, laboratory findings, treatment and outcome data were retrieved from the patients’ records. This study was approved by the Ethics Committee of the Shanghai Public Health Clinical Center (protocol code: Public Health 2022-s030-02). Due to this emergency public health event, written informed consent was waived and the patients included in this study provided their oral consent.

### 2.3. Related Definitions

According to China’s Novel Coronavirus Pneumonia Diagnosis and Treatment Plan (Seventh Edition) [15], the clinical classifications are mild, moderate, severe and critical. Mild type: the clinical symptoms are mild, with no manifestations of pneumonia on imaging. Moderate type: the patients have fever, respiratory tract symptoms, and other symptoms; imaging can show signs of pneumonia. Severe COVID-19 patients were defined as having oxygen saturation <93% on room air, dyspnea, respiratory rate ≥30/min, arterial oxygen partial pressure/fraction of inspired oxygen ratio <300 mmHg, and/or lung infiltrates >50% within 24–48 h. Critical COVID-19 patients were defined as having respiratory failure, septic shock, and/or multiple organ dysfunction or failure. 

Bacteriologically confirmed tuberculosis was defined as TB diagnosed in a biological specimen by a nucleic acid amplification test (GeneXpert, Cepheid), smear microscopy, or culture.

Clinically diagnosed tuberculosis was defined as the lack of bacteriological confirmation and the presence of at least 2 of the following:

1. Symptoms/signs suggestive of tuberculosis;
2. Chest radiograph consistent with tuberculosis;
3. Close tuberculosis exposure or immunologic evidence of mycobacterium tuberculosis infection;
4. Positive response to tuberculosis treatment.

### 2.4. RT-PCR Assay for SARS-CoV-2 RNA

Nasopharyngeal swabs were collected daily for detection of SASR-CoV-2 RNA from the fourth day after admission. Ct value (cycle threshold value) <= 35 for both ORF1ab and N gene was considered SARS-CoV-2 RNA positive. The viral shedding duration was defined as being from the date of the first positive test of SARS-CoV-2 RNA to the date of the first negative test in two consecutive samples.

### 2.5. Statistical Analysis

The data were presented as means ± SD or median (IQR) and comparisons were performed using *t*-tests or non-parametric tests. The categorical variables were presented as numbers and percentages and were compared by χ^2^ or Fisher’s exact test. The duration of SARS-CoV-2 shedding was examined by Kaplan–Meier survival analysis and log-rank test. A Cox proportional hazard model was used to identify risk factors associated with viral shedding. In multivariable-adjusted Cox regression models, the HR was adjusted for variables with *p* < 0.05 in the univariate analysis. A hazard ratio (HR) > 1 indicates prolonged SARS-CoV-2 RNA shedding. All analyses were performed using SPSS 26.0. The figures were constructed using Graphpad Prism 9.4. *p* < 0.05 was considered statistical significance.

## 3. Results

### 3.1. Clinical Characteristics of COVID-19 Patients with and without Tuberculosis

A total of 223 COVID-19 patients were enrolled, including 34 with tuberculosis and 189 without tuberculosis. The demographic and clinical characteristics were generally similar between COVID-19 patients with and without tuberculosis (Table 1).

For all of these, the median age was 47.0 years old (IQR, 32.0–65.0), the median body mass index (BMI) was 22.2 (IQR, 20.6–24.5), and the percentage of males was 64.6% (144/223). In total, 31.8% of the patients (71/223) had at least one comorbidity, and the most common comorbidities were diabetes (15.7%, 35/223) and hypertension (9.4%, 21/223), followed by cardio-cerebrovascular disease (5.8%, 13/223) and chronic obstructive pulmonary disease (COPD) (5.4%, 12/223). No HIV coinfection cases were found in this cohort. A total of 43.9% (98/223) had received a third booster dose of a COVID-19 vaccine. Nearly 80% of patients had symptomatic COVID-19, and the most common symptoms were fever, followed by cough and sputum. On admission, the median blood leukocyte count of patients was 5.5 × 10^9^/L (IQR, 4.3–7.2). A total of 30.9% (69/223) of patients developed lymphopenia (lymphocyte count <1.0 × 10^9^/L) and 20.6% (46/223) of them had an elevated C-reactive protein (CRP) level. 

Of the 34 COVID-19 patients with tuberculosis, 91.2% (31/34) had pulmonary tuberculosis (PTB), 20.6% (7/34) had extrapulmonary tuberculosis (EPTB), and 11.8% (4/34) had both PTB and EPTB. In total, 27 were laboratory-confirmed and 7 were clinical diagnosed tuberculosis patients.

### 3.2. Treatments and Outcomes of COVID-19 Patients with and without Tuberculosis

During hospitalization, 17 (7.6%, 17/223) patients required oxygen supply, 15 (6.7%, 15/223) patients received nirmatrelvir/ritonavir (Paxlovid), 6 (2.7%, 6/223) patients required high-flow ventilation, 3 (1.3%, 3/223) patients required invasive mechanical ventilation (IMV), 9 (4.0%, 9/223) patients were diagnosed as having severe or critical COVID-19, and 3 (1.3%, 3/223) patients died.

There was no significant difference between the COVID-19 patients with and without tuberculosis in the proportion of oxygen supply, nirmatrelvir/ritonavir use, high-flow ventilation and IMV requirements (O_2_ supply, 8.8% vs. 7.4%, *p* = 0.729; nirmatrelvir/ritonavir, 8.8% vs. 6.3%, *p* = 0.707; high-flow ventilation requirement, 5.8% vs. 2.1%, *p* = 0.228; IMV requirement, 2.9% vs. 1.1%, *p* = 0.393). Although the COVID-19 patients with tuberculosis had a higher proportion of severe/critical COVID-19 diagnosis and deaths (severe/critical COVID-19, 8.8% vs. 3.2%, *p* = 0.142; death, 2.9% vs. 1.1%, *p* = 0.393) (Table 2), no significant differences were seen between the COVID-19 patients with and without tuberculosis.

Of the three deaths, one (2.9%, 1/34) COVID-19 patient with tuberculosis, a 26-year-old male with no comorbidities, who was diagnosed with critical COVID-19 and severe tuberculosis (involving lung, pleura, intestine, peritoneum) at admission, required invasive ventilation and extracorporeal membrane oxygenation (ECMO). Three weeks after hospitalization, the patient died of septic shock and sepsis-related organ failure. Two (1.1%, 2/189) COVID-19 patients without tuberculosis were diagnosed with severe COVID-19 at admission, and one was diagnosed with critical COVID-19 at discharge. One died of ARDS (acute respiratory distress syndrome) during chemotherapy for lung cancer, and the other of sudden death during hemodialysis.

### 3.3. Risk Factors for SARS-CoV-2 Shedding 

**Figure 1:**
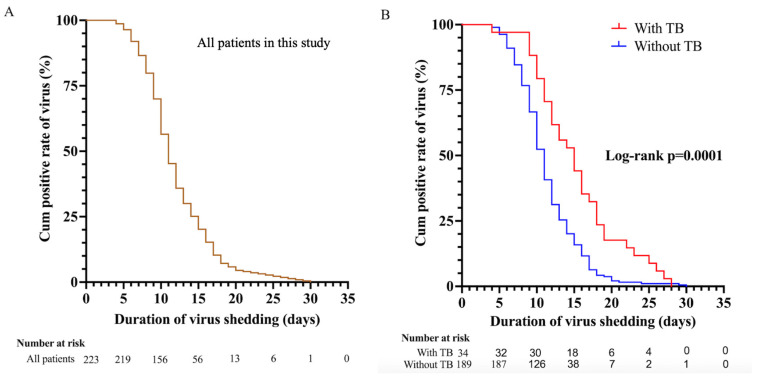
(**A**,**B**), Kaplan–Meier plot for the time from the first positive test to the first day of nucleic acid Ct value >35 for both ORF 1ab and N gene among all patients (n = 223). (**A**) All patients in this study. (**B**) 34 patients with tuberculosis and 189 patients with non-tuberculosis. Ct, cycle threshold; ORF 1ab, open reading frame 1ab; N gene, nucleocapsid.

In all patients, the median duration of SARS-CoV-2 RNA shedding was 11.0 days (IQR, 9.0–15.0) (Figure 1A), while the median duration of viral shedding in patients without tuberculosis and with tuberculosis was 11.0 days (IQR, 9.0–14.0) and 15.0 days (IQR, 11.0–18.2), respectively (*p* = 0.0001) (Figure 1B).

We further explored SARS-CoV-2 shedding duration and potential risk factors. Cox univariate analysis showed that having tuberculosis (HR = 1.93, 95% CI 1.33–2.80; *p* = 0.001), being of elderly age (HR = 1.02, 95% CI 1.01–1.03; *p* = 0.000), being male (HR = 1.54, 95% CI 1.17–2.04; *p* = 0.003), having comorbidities (HR = 1.48, 95% CI 1.11–1.97; *p* = 0.007), and being diagnosed with severe or critical COVID-19 (HR = 5.67, 95% CI 2.42–13.25; *p* = 0.000) were significant predictors of prolonged SARS-CoV-2 RNA detection, whereas the use of Paxlovid (HR = 0.47, 95% CI 0.27–0.83; *p* = 0.009) was a predictor of shortened SARS-CoV-2 RNA detection. In a multivariate Cox proportional hazard regression model, having tuberculosis (HR = 2.21, 95% CI 1.37–3.00; *p* = 0.000), being of elderly age (HR = 1.02, 95% CI 1.01–1.03; *p* = 0.001) and being diagnosed with severe or critical COVID-19 (HR = 5.63, 95% CI 2.10–15.05; *p* = 0.001) were independent factor associated with a prolonged virus time of SARS-CoV-2 (Table 3).

### 3.4. Subgroup Analysis of Severity of Hospitalization and SARS-CoV-2 Shedding Duration 

To further explore whether the duration of anti-tuberculosis treatment has an effect on the severity of COVID-19 patients with tuberculosis and the clearance of nucleic acids, we classified the duration of anti-tuberculosis treatment as less than 2 months (intensive phase), 2–4 months (early consolidation phase), and more than 4 months (late consolidation phase).

**Figure 2:**
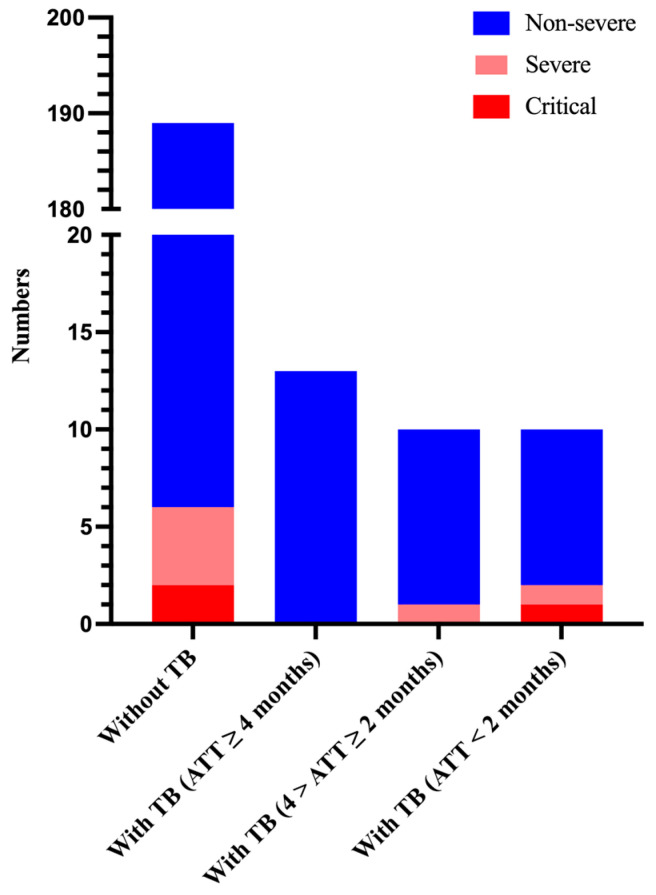
Number of patients and their severity of hospitalization on the basis of anti-tuberculosis therapy duration. ATT, anti-tuberculosis therapy.

We found that patients without tuberculosis (3.2%, 6/189) and patients with tuberculosis receiving anti-tuberculosis therapy (ATT) for ≥ 4 months (0%, 0/13) were less likely to have a progression of COVID-19 into a severe and critical stage. However, among these patients who were receiving ATT for < 2 months, two patients (20%, 2/10) were discharged with a diagnosis of severe or critical COVID-19 (one with severe COVID-19 and one with critical COVID-19). In addition, among the patients who were receiving ATT (4 months > ATT ≥ 2 months), one patient (10%, 1/10) was diagnosed with severe COVID-19 during hospitalization (Figure 2).

**Figure 3:**
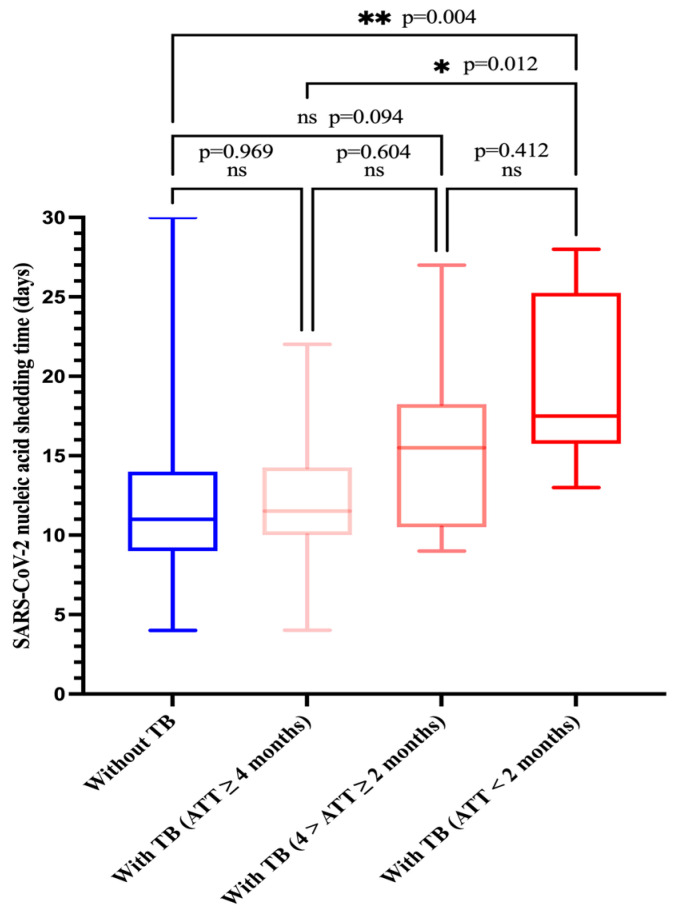
Comparison of SARS-CoV-2 nucleic acid shedding time by four groups. Group 1: Without TB, group 2: with TB (ATT ≥ 4 months), group 3: with TB (4 months > ATT ≥ 2 months), group 4: with TB (ATT < 2 months). ATT, anti-tuberculosis therapy.

Among the 34 tuberculosis patients (tuberculosis group), 10 patients would receive or were already receiving anti-tuberculosis therapy (ATT) (ATT < 2 months) at the time of admission and their SARS-CoV-2 RNA clearance was 17.5 days (IQR, 15.8–25.2); 10 patients were receiving ATT (4 months > ATT ≥ 2 months) and their viral RNA clearance was 15.5 days (IQR, 10.5–18.3); and 14 patients were receiving ATT (ATT ≥ 4 months) and their SARS-CoV-2 shedding duration was 11.5 days (IQR,10.0–14.3). A total of 189 patients did not have tuberculosis (non-tuberculosis group) and their SARS-CoV-2 shedding duration was 11.0 days (IQR, 9.0–14.0). Patients who would receive or were receiving ATT for < 2 months had a significantly longer duration of virus shedding as compared to those who did not have tuberculosis or were receiving ATT for ≥ 4 months (median duration of virus shedding: 17.5 days vs. 11.0 days, *p* = 0.004; 17.5 days vs. 11.5 days, *p* = 0.012), but this was not observed in those who were receiving ATT (4 months > ATT ≥ 2 months) (17.5 days vs. 15.5 days, *p* = 0.412) (Figure 3). In addition, SARS-CoV-2 RNA shedding duration was similar in both the non-tuberculosis group and the tuberculosis group (ATT ≥ 4 months) (11 days vs. 11.5 days, *p* = 0.969). The SARS-CoV-2 RNA shedding duration of patients who did not have tuberculosis was shorter than that of those of who were receiving ATT (4 months > ATT ≥ 2 months), but there was no significant difference (11 days vs. 15.5 days, *p* = 0.094).

## 4. Discussion

In this retrospective cohort study, we analyzed the differences in the clinical characteristics and SARS-CoV-2 shedding duration between COVID-19 patients with and without tuberculosis. 

Our study showed that a higher proportion of COVID-19 patients with tuberculosis require high-flow ventilation and IMV but did not have a significantly higher rate of severe morbidity and mortality than those without tuberculosis. This result is not consistent with the findings (tuberculosis was significantly associated with disease severity and poor prognosis in COVID-19 patients) of previous meta-analyses and studies [9,10,11,16,17]. Some reasons may explain these results. Firstly, our sample was small. Secondly, the two meta-analyses and two studies both showed fatality rates of coinfection above 10% [9,10,17,18], whereas the fatality rate of coinfection was 2.9% in our study; a possible explanation for this is that the treatment regimens for coinfected patients were highly heterogeneous. Few of the articles included in these meta-analyses provided a detailed description of the tuberculosis treatment of coinfected patients, and very little attention was paid to the prognostic impact of coinfected patients’ duration of anti-tuberculosis treatment. However, in our study, only 29.4% (10/34) of coinfected patients received ATT for < 2 months, and most of the coinfected patients (70.6%, 20/34) completed the intensive phase of anti-tuberculosis treatment; a higher proportion of completion of intensive anti-tuberculosis treatment may be a key reason for lower mortality in coinfected patients and the narrowing of the difference in mortality between the two groups. In addition, an early observational study from China showed that patients with active TB or latent TB were not only potentially more susceptible to SARS-CoV-2 infection, but COVID-19 disease may be more severe and also progress more rapidly [19]. Although this study was small with only 13 SARS-CoV-2 and Mtb coinfected cases, these findings were later supported by studies which surmised that previous TB and current TB were associated with increased COVID-19-related deaths and were an independent risk factor for mortality [20,21,22]. Therefore, an effective anti-TB treatment provides a better control of tuberculosis, facilitates immune recovery, and probably reduces mortality in SARS-CoV-2 and Mtb coinfected patients. Conversely, a prospective, multicountry study showed that 788 patients with COVID-19 and TB (active or sequalae) who were diagnosed with COVID-19 after the end of the TB treatment appeared to have a poorer prognosis, with a higher number and proportion of “non-recovery” COVID-19 cases [23].

The viral load and duration of viral shedding could serve as potential markers to assess the infectivity, transmissibility, patient isolation decisions and curative efficacy. In this study, we investigated the shedding dynamics of SARS-CoV-2 in patients with COVID-19 and tuberculosis coinfection. Our data showed that tuberculosis in patients was an independent factor associated with a prolonged duration of viral shedding. One possible explanation was that patients with Mtb and SARS-CoV-2 coinfection have a decreased total lymphocyte count, SARS-CoV-2-specific CD4+ T cells and responsiveness to SARS-CoV-2 antigens compared to patients with COVID-19 alone [24]. Additionally, age was an independent factor associated with prolonged SARS-CoV-2 RNA shedding, which is consistent with the findings of previous studies [25,26]. Some studies also illustrated that senior age was also a risk factor of severe COVID-19 and there was an age-dependent enhancement in severe COVID-19 and cytokine storm syndromes [27,28], and an important case report suggested that the reason for the link between senior age and severe COVID-19 might be respiratory flora imbalance in the lower respiratory tract [29], which may affect viral clearance. Moreover, severe or critical diseases were found to be important independent risk factors for prolonged viral RNA shedding duration. The results indicated that viral RNA shedding was associated with disease severity. Similarly, severe patients had more prolonged MERS-CoV shedding in the severe group than in the mild group [30], and this can be explained by the kinetic analysis of viral RNA shedding (being detected longer, being more sustained and at higher levels in lower respiratory tract specimens than in upper respiratory tract specimens [31,32,33]). However, our results showed that booster vaccination did not appear to shorten SARS-CoV-2 RNA shedding compared with one-dose or two-dose primary vaccination. This result may be related to the type of vaccine and timing of the vaccination. Most individuals in China had been vaccinated with an inactivated vaccine such as Corona Vac (SINOVAC Life Sciences Co., Ltd., Beijing, China) and Sinopharm BBIBP-CorV, while a higher humoral response was reported after using the mRNA vaccine compared with that observed with the inactivated vaccine [34,35,36]. There was also evidence of a decay in vaccine-induced neutralization titers during the first six months following the second dose [37]. Therefore, we hypothesize that booster vaccination may increase antibody titer in a short time, but the effect of the third dose would gradually decline. The use of paxlovid was not observed to accelerate the clearance of the viral load in our study, which may be related to a delayed paxlovid treatment (>5 days after diagnosis) [38]. In addition, age-related comorbidities might result in prolonged viral shedding among the elderly [39], but no significant difference was noted concerning comorbidities in this study, similar to previous studies [40,41]. The effect of comorbidities on the clearance of the viral load in COVID-19 patients with tuberculosis still needs further investigation. COVID-associated immunodepression may also stimulate the development of Pneumocystis jirovecii pneumonia (PJP), and either colonization or probable/possible Pneumocystis pneumonia can be determined by using a cutting-edge molecular diagnostic test [42]. An unbalanced distribution rate of pneumocystis jirovecii in COVID-19 patients with tuberculosis and COVID-19 patients without tuberculosis might lead to a different duration of virus shedding.

We also found that COVID-19 patients with tuberculosis who had received anti-tuberculosis treatment for 4 months or more (ATT ≥ 4 months) had a faster time to nucleic acid conversion than those who had not completed the intensive anti-tuberculosis treatment (ATT < 2 months). This might be closely related to the ability of effective anti-tuberculosis therapy to improve the host immune response, as (1) anti-tuberculosis chemotherapy rescues Th1 and CD8+ T effector levels in pulmonary tuberculosis patients [43] and (2) Silva et al. observed that CD4+ and CD8+ T cells’ activation and increased Th1 cytokine production like IFN-γ and TNF-α are associated with a clinical cure of tuberculosis [44]. Therefore, we have reason to believe that the effective control of tuberculosis can restore the body’s immunity and accelerate the clearance of the virus. In addition, a previous hypothesis has indicated that CD8+ T cells play a critical role in the antiviral response during acute viral infections, while B cells are responsible for the prevention of infection and eventual viral clearance [45], and a prospective cohort study with a small sample size demonstrated that prolonged shedding of SARS-CoV-2 in immunocompromised patients was significantly associated with the level of the neutralizing antibody response [46].

To the best of our knowledge, this is the first study to focus on the duration of viral shedding and related factors in COVID-19 patients with tuberculosis. We found that tuberculosis in patients was a risk factor for a prolonged duration of viral shedding, and the duration of viral shedding was shortened in patients receiving or who had received effective anti-tuberculosis therapy. In addition, we also found that COVID-19 patients with tuberculosis were prone to have more severe disease and a worse prognosis. Our findings suggest that special attention and care would be beneficial for COVID-19 patients with tuberculosis, especially those patients with tuberculosis who have not received effective anti-tuberculosis therapy or have received anti-tuberculosis therapy for less than 2 months.

Our study also has limitations. First, our sample size of COVID-19 patients with tuberculosis is small. Second, we do not provide cycle threshold (Ct) values and cannot verify whether the viral load of patients with tuberculosis differs from that of patients treated for tuberculosis in the early stages of infection, which may also affect the duration of viral shedding. Third, we do not analyze the effect of viral strains such as the Delta variant or Omicron variant on disease severity and viral clearance in COVID-19 patients with tuberculosis. Fourth, we lack laboratory immunological results (such as the monitoring of SARS-CoV-2 neutralizing antibody levels, detection of T cell phenotype and function, and measurement of chemokine) to further understand and analyze the interaction between SARS-CoV-2 and tuberculosis, and its impact on patients after coinfection.

## 5. Conclusions

COVID-19 patients with tuberculosis tend to have more severe disease and a worse prognosis. Patients with tuberculosis have a prolonged viral shedding duration. Thus, tuberculosis–COVID-19 coinfection patients require special attention and care, especially those who have received anti-tuberculosis therapy for less than 2 months. Effective tuberculosis control can help shorten the duration of viral shedding.

## Figures and Tables

**Table 1:** The demographic and clinical characteristics of the study patients.

	Overall (n = 223)	With TB (n = 34)	Without TB (n = 189)	*p* Value
Age (years)	47.0 (32.0–65.0)	49.0 (31.8–59.8)	47.0 (32.0–65.0)	0.479
Male (n, %)	144 (64.6%)	26 (76.5%)	118 (62.4%)	0.115
BMI	22.2 (20.6–24.5)	21.8 (21.0–23.4)	22.3 (20.6–24.6)	0.428
Comorbidities (%)	71 (31.8%)	13 (38.2%)	58 (30.7%)	0.384
Diabetes	35 (15.7%)	8 (23.5%)	27 (14.3%)	
Hypertension	21 (9.4%)	3 (8.8%)	18 (9.5%)	
COPD	12 (5.4%)	2 (5.9%)	10 (5.3%)	
Cardio-cerebrovascular disease	13 (5.8%)	2 (5.9%)	11 (5.8%)	
Chronic kidney disease	9 (4.0%)	2 (5.9%)	7 (3.7%)	
Cancer	3 (1.3%)	0 (0)	3 (1.6%)	
Autoimmune disease	2 (0.9%)	0 (0)	2 (1.1%)	
Vaccination booster				
Yes	98 (43.9%)	18 (52.9%)	80 (42.3%)	0.251
TB location ^a^				
PTB	/	31 (91.2%)	/	/
EPTB	/	7 (20.6%)	/	/
Clinical presentation on admission				0.597
Asymptomatic	45 (20.2%)	8 (23.5%)	37 (19.6%)	
Symptomatic	178 (79.8%)	26 (76.5%)	152 (80.4%)	
Laboratory findings on admission				
Blood leukocyte count (3.5–9.5 × 10^9^/L)	5.5 (4.3–7.2)	5.4 (4.2–7.1)	5.6 (4.3–7.2)	0.724
Lymphopenia ^b^ (%)	69 (30.9%)	15 (44.1%)	54 (28.6%)	0.071
C-reactive protein ≥ 10 mg/L (%)	46 (20.6%)	10 (29.4%)	36 (19.0%)	0.169

BMI, body mass index; COPD, chronic obstructive pulmonary diseases; PTB, pulmonary tuberculosis; EPTB, extra-pulmonary tuberculosis. ^a^ Four patients had both pulmonary and extrapulmonary TB; ^b^ lymphocyte count <1.0 × 10^9^/L.

**Table 2:** Treatments and outcomes of the study patients.

	Overall (n = 223)	With TB (n = 34)	Without TB (n = 189)	*p* Value
O_2_ supply (%)	17 (7.6%)	3 (8.8%)	14 (7.4%)	0.729 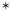
Nirmatrelvir/ritonavir (%)	15 (6.7%)	3 (8.8%)	12 (6.3%)	0.707 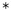
Required high-flow ventilation	6 (2.7%)	2 (5.8%)	4 (2.1%)	0.228 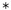
Required IMV (%)	3 (1.3%)	1 (2.9%)	2 (1.1%)	0.393 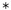
COVID-19 diagnosis				0.142 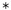
Severe or critical	9 (4.0%)	3 (8.8%)	6 (3.2%)	
Non-severe 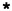	214 (96.0%)	31 (91.2%)	183 (96.8%)	
Die (%)	3 (1.3%)	1 (2.9%)	2 (1.1%)	0.393 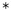

IMV, invasive mechanical ventilation.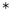 Fisher’s exact test; 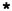 non-severe include mild and moderate.

**Table 3:** Univariate analysis according to the log-rank test and multivariate analysis with a Cox proportional hazard model regarding the viral shedding duration in COVID-19 patients with tuberculosis.

Factors	Univariate Analysis		Multivariate Analysis	
	HR (95% CI)	*p*	HR (95% CI)	*p*
With TB vs. without TB	1.927 (1.325–2.803)	**0.001**	2.207 (1.370–2.999)	**0.000**
Age (years)	1.017 (1.009–1.025)	**0.000**	1.018 (1.007–1.030)	**0.001**
Gender				
Male vs. female	1.543 (1.165–2.044)	**0.003**	1.083 (0.799–1.468)	0.608
BMI	1.006 (0.960–1.053)	0.806		
Comorbidities				
One or more vs. none	1.481(1.114–1.967)	**0.007**	1.166 (0.790–1.720)	0.439
Vaccination booster				
Yes vs. no	0.927 (0.710–1.209)	0.575	0.922 (0.703–1.210)	0.558
Treatment				
Paxlovid (required vs. not required)	0.473 (0.271–0.827)	**0.009**	0.823 (0.432–1.571)	0.555
Disease severity				
Severe or critical vs. non-severe 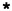	5.667 (2.424–13.249)	**0.000**	5.625 (2.103–15.046)	**0.001**

BMI, body mass index, 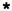 non-severe includes mild and moderate.

## Data Availability

All data reported in the manuscript will be made available upon request to the corresponding author.
